# Aerodynamics of the newly approved football for the English Premier League 2020–21 season

**DOI:** 10.1038/s41598-021-89162-y

**Published:** 2021-05-05

**Authors:** Takeshi Asai, Sungchan Hong

**Affiliations:** Faculty of Health and Sports Sciences, University of Tsukuba, Tsukuba, 305-8574 Japan

**Keywords:** Engineering, Physics

## Abstract

Footballs are typically constructed with 32 panels. Recently, the number of panels has been successively reduced to 14, 8, and 6 panels, and official balls have been adopted with complex panel shapes and aerodynamics that differ from those of 32-panel balls. The official ball for the 2020–21 season of the English Premier League comprises just four panels with a complex panel shape and surface groove design; however, its aerodynamics have not yet been clarified. This study aims to clarify the aerodynamic characteristics (drag, side force, lift force, their deviations, and critical Reynolds number) of the new 4-panel ball (Flight 2020, Nike) in comparison to a 6-panel ball (Tsubasa 2020, Adidas) and conventional 32-panel ball (Pelada 2020, Molten) using a wind tunnel test, surface design measurement, and a simple 2D flight simulation. The results showed that Flight 2020 has greater surface roughness and smaller critical Reynolds number than Pelada 2020 and Tsubasa 2020, resulting to its marginally greater drag force in the supercritical region, and slightly smaller fluctuations of the side and lift forces. Furthermore, Flight with a symmetrical orientation exhibits a significantly higher drag coefficient in the supercritical region, suggesting its greater air resistance during flight under this condition.

## Introduction

The trajectory of a sports ball is greatly influenced by its aerodynamics^1^. Many studies have focused on the aerodynamics of balls for baseball^2–4^, football^5,6^, golf^7,8^, volleyball^9,10^, tennis^11,12^, and other sports^13^. For footballs, their aerodynamic characteristics, such as drag, side force, lift force, and critical Reynolds number, have been analysed mainly through wind tunnel tests^14,15^. In particular, the critical Reynolds number associated with the drag crisis is a measure of the surface roughness of the ball and is affected by the number of ball panels and their shape^16,17^. Furthermore, the numerical simulations of the flight trajectory have shown that trajectory varies depending on the number and shape of the panels^18–20^. In addition, it is reported that the fluctuations in the unsteady side and lift forces during the slow rotation of the ball (i.e. knuckling or wobbling)^21,22^ are also affected by the number and shape of the ball panels^23,24^.

Conventionally, footballs have 32 panels comprising 12 pentagonal panels and 20 hexagonal panels. In recent years, balls with fewer panels and complex panel shapes have been adopted as official balls, such as the 14-panel Teamgeist 2006 (Adidas), 8-panel Jabulani 2010 (Adidas), 6-panel Brazuca 2014 (Adidas), and 6-panel Telstar18 2018 (Adidas). As the number of ball panels decreases from 32 to 14 and 8, their critical Reynolds numbers tend to increase from ~ 2.5 × 10^5^ to ~ 3.1 × 10^5^ and to ~ 3.6 × 10^5^, respectively^25–27^. However, when the number of ball panels is further reduced to 6, the critical Reynolds number tends to decrease to ~ 2.3 × 10^5^ and 2.7 × 10^5^. The aerodynamics of the new 4-panel ball named Flight 2020 (Nike), which is the official ball of the 2020–21 season of the English Premier League has yet to be clarified.

In this regard, this study aims to clarify the aerodynamic characteristics (drag, side force, lift force, their deviations, and critical Reynolds number) of the new 4-panel ball (Flight 2020, Nike), in comparison to a 6-panel ball (Tsubasa 2020, Adidas) and conventional 32-panel ball (Pelada 2020, Molten) using a wind tunnel test, surface design measurement, and simple 2D flight simulation.

## Methods

### Wind tunnel test

**Figure 1:**
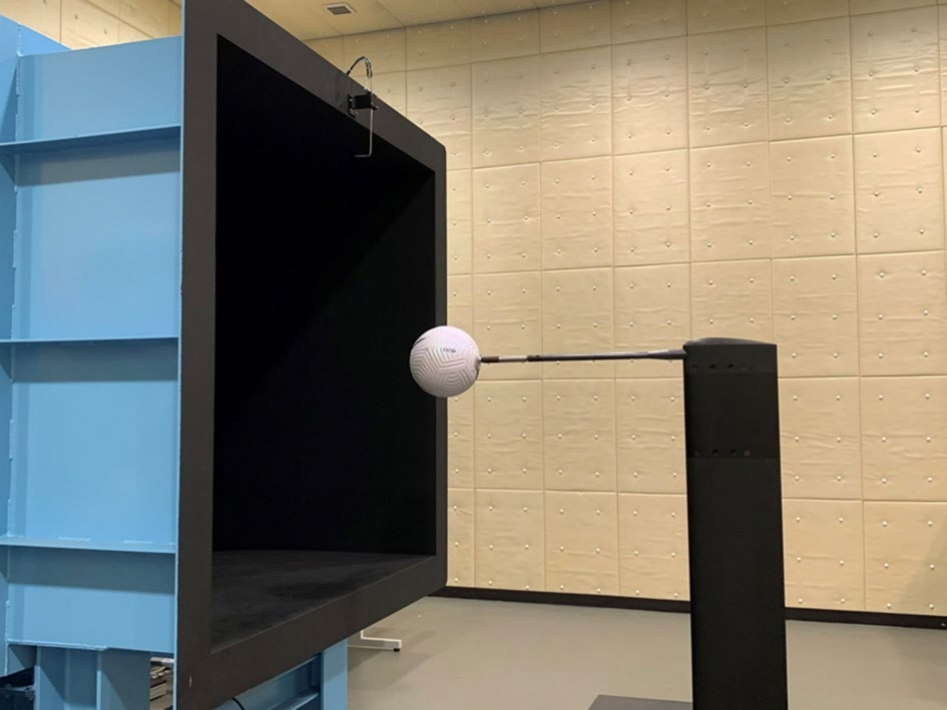
Setup of the circulation-type low-speed low-turbulence wind tunnel at the University of Tsukuba.

To obtain the basic aerodynamic characteristics of the ball, a wind tunnel test was conducted. A circulation-type low-speed low-turbulence wind tunnel (San Technologies Co., Ltd.) at the University of Tsukuba was used for the experiment (Fig. 1). This wind tunnel has a maximum wind speed of 55 m/s, nozzle size of 1.5 × 1.5 m, wind speed distribution of ± 0.5%, and degree of turbulence of less than 0.1%. The blockage of the measured football was within 5% of the nozzle size. Since turbulence intensity is known to affect the transition velocity^28,29^, the turbulence intensity (< 0.1%) of this experiment is presumed to be slightly smaller than that of an actual football stadium^30,31^. However, to obtain the basic aerodynamic characteristics of the ball, the experiment was conducted using a wind tunnel setup. The wind speed was incremented from 7 m/s (Re ≈ 1.0 × 10^5^) to 35 m/s (Re ≈ 5.0 × 10^5^) in intervals of 1 m/s every 10 s (100 Hz). The main flow speed was continuously controlled using a computer, and the aerodynamic force applied to the ball was measured for 10 s after the main flow reached the set flow speed and became sufficiently stable.

The forces acting on the footballs were measured with a sting-type six-component force measurement balance (LMC-61256, Nissho Electric Works). The measured aerodynamic drag force (*D*), side force (*S*), and lift force (*L*) were converted to the drag force coefficient (*C*_*d*_), side force coefficient (*Cs*), and lift force coefficient (*Cl*), respectively:

1 $$C_{d} { } = { }\frac{2D}{{\rho U^{2} A}}$$

2 $$C_{s} { } = { }\frac{2S}{{\rho U^{2} A}}$$

3 $$C_{l} { } = { }\frac{2L}{{\rho U^{2} A}}$$

where *ρ* is the air density (*ρ* = 1.2 kg/m^3^), *U* is the flow speed, and *A* is the projected area of the football (*A* = *π* × 0.11^2^ = 0.038 m^2^).

The back of the ball was supported by a sting with a length of 0.8 m and width of 0.02 m in the wind tunnel. Since placing the six-component force measurement balance behind the football reduced the effect of the sting vibrations to a negligible value, the magnitude of the sting forces was neglected.

Three types of balls were used in this experiment: the new 4-panel ball (Flight 2020, Nike), a 6-panel ball (Tsubasa 2020, Adidas), and a conventional 32-panel ball (Pelada 2020, Molten). Tsubasa 2020 was the official ball of the 2020 FIFA U-20 Women’s World Cup and Pelada 2020 is a conventional ball approved by FIFA. We defined two faces for the ball orientation: (A) symmetric (about the horizontal axis) and (B) asymmetric (rotated 30° horizontally, clockwise from the top view). We then measured the aerodynamic forces acting on each ball face. The symmetric orientation cases were designated as Pelada A, Tsubasa A, and Flight A, and the asymmetric orientation cases were designated as Pelada B, Tsubasa B, and Flight B. In order to examine the accuracy of the measurement balance and validity of the experiment, we also measured the drag coefficient of a smooth sphere made of plastic (radius is 0.11 m).

### Tested footballs

For the statistical analysis, we employed a multiple comparison test (Tukey–Kramer method) to compare the average drag coefficients and average standard deviation of the side and lift coefficients in the supercritical region.

### Panel shape and surface groove design measurement

To study the characteristics of the football surface shape in more detail, the surface shape parameters that affect the surface roughness were measured. The parameters obtained in this study were the length of the panel seams (bonds and grooves) and the width and depth of the grooves. The length of the panel joints was measured using a curvimeter (Concurve 10; Koizumi Sokki Mfg. Co., Ltd., Japan). The width and depth of the grooves were measured using a high-speed 2D laser scanner (LJ-V7000; Keyence Corp., Japan). To measure these parameters, all the grooves of the football were covered using clay, with the height of the imprint representing the panel groove depth and the width representing the panel groove width.

### Ball trajectory simulation

We conducted a simple 2D flight simulation to compare the effects of the drag coefficients and orientations on the flight distance and trajectory of the different balls in the subcritical regime (*U* = 15 m/s) and supercritical regime (*U* = 30 m/s). For all cases, the initial flight trajectory was set to an angle of 30°^18^.

The following equations were used for the simulation:

4 $$ma_{h} = { } - D\cos \gamma$$

5 $$ma_{v} = { } - D\sin \gamma - mg$$

where *m* is the mass of the football, $a_{h}$ is the horizontal acceleration of the ball, $a_{v}$ is the vertical acceleration of the ball, $g$ is the gravitational acceleration, and $\gamma$ is the initial attack angle of the ball flight trajectory.

The 2D ball velocity and displacement were obtained by the explicit Euler method by interpolating the drag coefficient at each time step (0.001 s) using a sixth-degree polynomial approximation^23,25^. The lift and side forces acting on the ball were omitted in this ball trajectory simulation.

We employed a multiple comparison test (Tukey–Kramer method) to compare the average standard deviations of the side and lift forces at the initial ball (flow) speeds of 15 and 30 m/s (Re of ~ 2.1 × 10^5^ and ~ 4.2 × 10^5^, respectively).

## Results

### Drag coefficient in the wind tunnel

**Figure 2:**
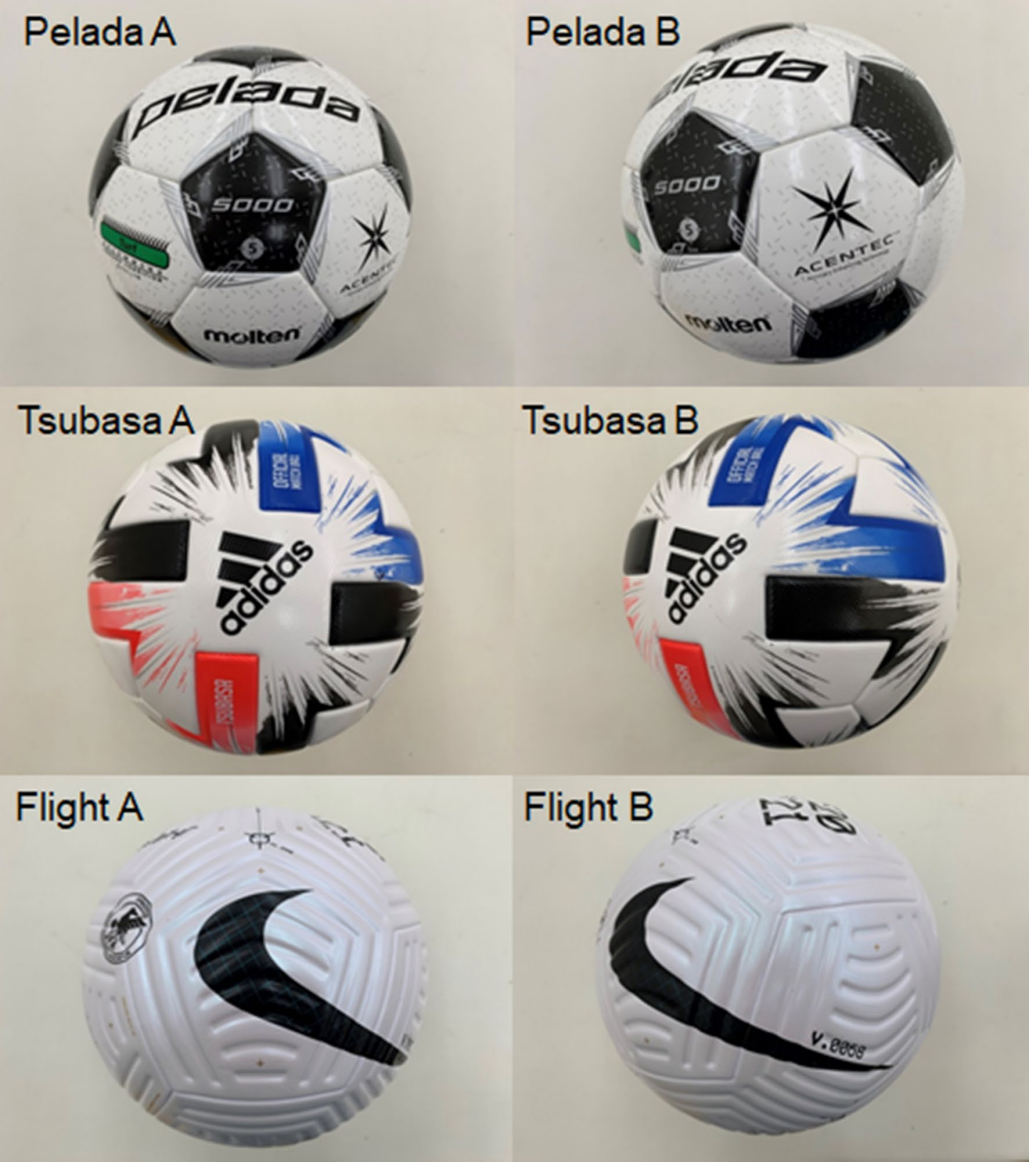
Footballs used for the test and their panel orientations: Pelada A and Pelada B (32 panels, Molten), Tsubasa A and Tsubasa B (6 panels, Adidas), and Flight A and Flight B (4 panels, Nike). A indicates a symmetric orientation while B indicates an asymmetric orientation).

**Figure 3:**
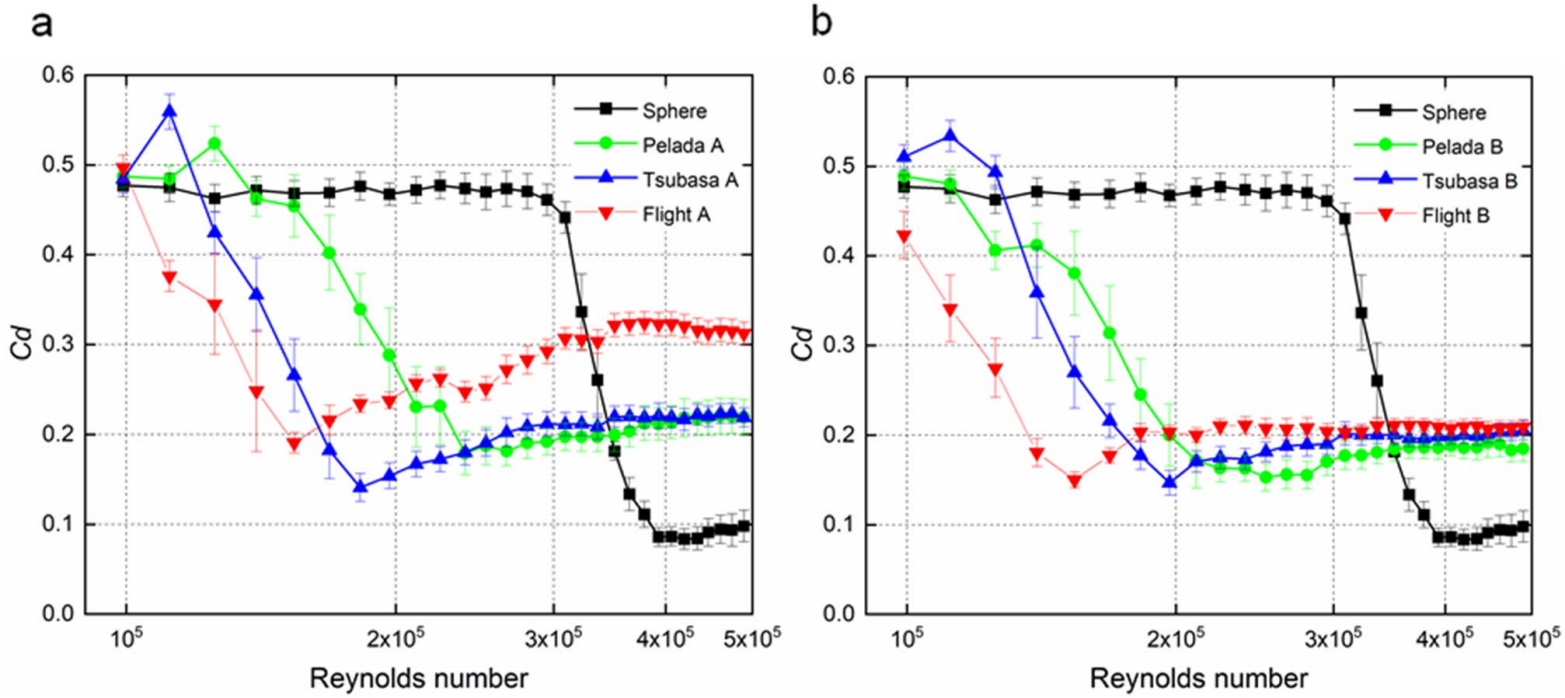
Drag coefficient versus Reynolds number for each type of ball and panel orientation: (**a**) symmetric and (**b**) asymmetric orientations.

Wind tunnel experiments (Fig. 1) were performed with Flight 2020, Tsubasa 2020, and Pelada 2020 (Fig. 2). A drag crisis was observed in the drag coefficient (*C*_*d*_) for all balls, while the critical Reynolds number varied with the ball and orientation (Fig. 3). The standard deviations of *Cd* are indicated as error bars in the figure. The influence of this deviation on the flight distance is approximately 0.8% for Flight A (initial speed of 30 m/s and attack angle of 30°), which is smaller than the difference in the flight distance due to the ball type and face orientation of this study. The drag coefficient of the smooth sphere in this study is approximately 0.47 and 0.10 in the subcritical and supercritical region, respectively, and the critical Reynolds number is approximately 4.0 × 10^5^.

For the symmetric orientation cases, the critical Reynolds numbers are ~ 2.39 × 10^5^ (*C*_*d*_ ≈ 0.179) for Pelada A, ~ 1.82 × 10^5^ (*C*_*d*_ ≈ 0.141) for Tsubasa A, and ~ 1.54 × 10^5^ (*C*_*d*_ ≈ 0.191) for Flight A. Meanwhile, for the asymmetric orientation cases, the critical Reynolds numbers are ~ 2.52 × 10^5^ (*C*_*d*_ ≈ 0.153) for Pelada B, ~ 1.97 × 10^5^ (*C*_*d*_ ≈ 0.148) for Tsubasa B, and ~ 1.54 × 10^5^ (*C*_*d*_ ≈ 0.150) for Flight B. For both the symmetric and asymmetric orientations, the balls with a smaller critical Reynolds number tend to have a larger *C*_*d*_ in the supercritical region.

In the supercritical region, the average drag coefficients are ~ 0.19 (s.d. = 0.017) for Pelada 2020, ~ 0.20 (s.d. = 0.017) for Tsubasa 2020, and ~ 0.25 (s.d. = 0.049) for Flight 2020. Thus, the average drag coefficients of Flight 2020 in the supercritical region are significantly larger than those of Pelada 2020 and Tsubasa 2020 (p < 0.001).

### Extended total length of the panel seams (bonds and grooves) on the ball surface

The extended total length of the panel seams (bonds and grooves) on the ball surface are ~ 10,910 mm for Flight 2020, which is longer than that of the other balls (Table 1). Flight 2020 has a groove depth of 0.7 mm, which is similar to that of the other balls, and a width of 5.7 mm, which is substantially wider than that of the other balls.

**Table 1:** Extended total length of the panel seam (bonds and grooves), groove depth, and groove width on the ball surface.

	Total seam length (mm)	Depth (mm)	Width (mm)
Pelada 2020	4050	0.8	3.4
Tsubasa 2020	4330	1.0	3.8
Flight 2020	10910	0.7	5.7

The correlation coefficients of the critical Reynolds number to the extended total length, groove width, and groove depth are 0.81 (*p* = 0.052), 0.87 (*p* = 0.023), and 0.20 (*p* = 0.702) respectively in the symmetric and asymmetric orientation cases of the three types of balls.

### Ball trajectory simulation

**Figure 4:**
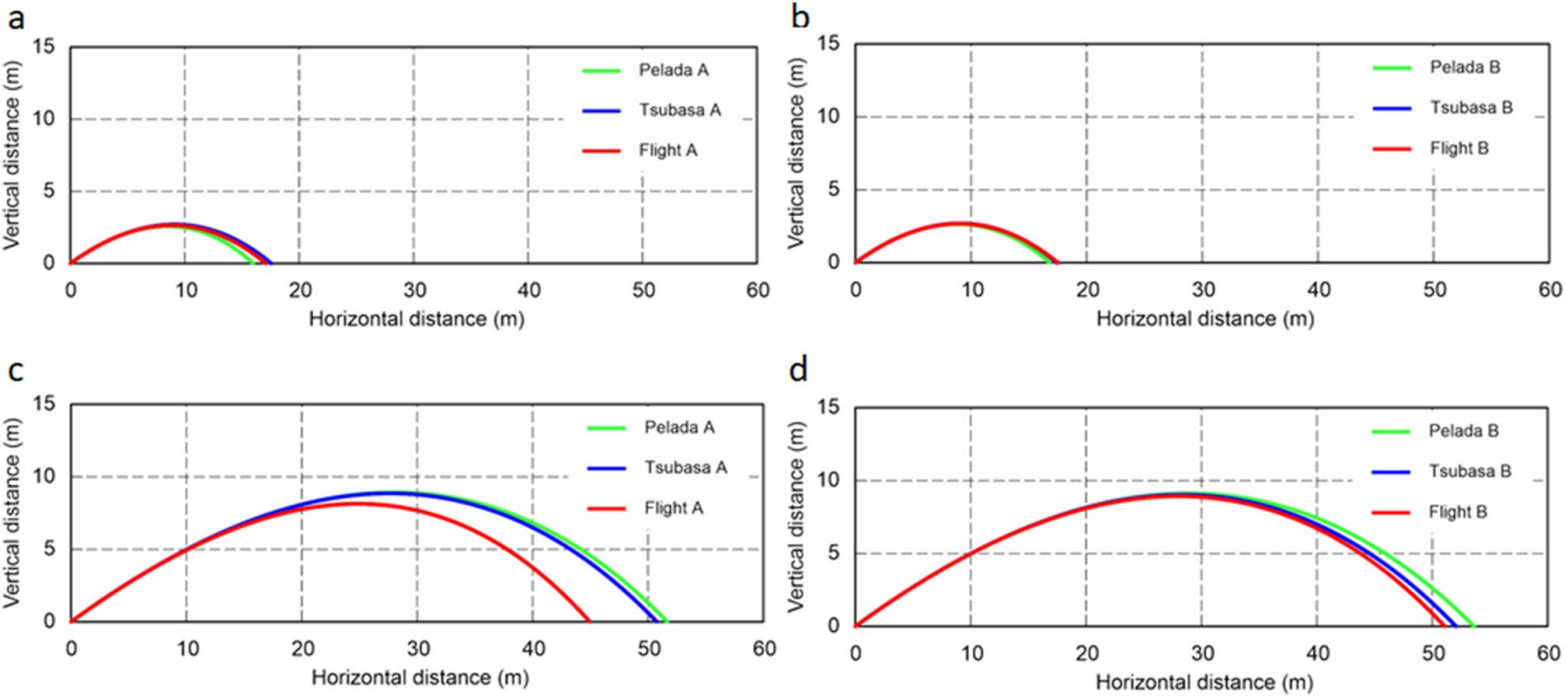
Comparison of the flight trajectories for Flight 2020, Tsubasa 2020, and Pelada 2020: (**a**) symmetric and (**b**) asymmetric orientations in the critical regime (initial ball speed of 15 m/s; Re = 2.1 × 10^5^); (**c**) symmetric and (**d**) asymmetric orientations in the supercritical regime (initial ball speed of 30 m/s; Re = 4.2 × 10^5^).

For the symmetric orientation cases at an initial ball speed of 15 m/s, Tsubasa A has the longest flight distance at 17.6 m, followed by Flight A at 17.1 m and Pelada A at 16.0 m (Fig. 4). Meanwhile for the asymmetric orientation cases at an initial ball speed of 15 m/s, Tsubasa B has the longest flight distance at 17.6 m, followed by Flight A at 17.5 m, and Pelada B at 16.8 m. When the initial ball speed is 30 m/s, Pelada A has the longest flight distance at 51.7 m, followed by Tsubasa A at 50.8 m and Flight A at 45.0 m among the symmetric orientation cases. In contrast, for the asymmetric orientation cases with an initial ball speed of 30 m/s, Pelada B has the longest flight distance at 53.6 m, followed by Tsubasa B at 52.1 m and Flight B at 51.1 m.

### Fluctuation of side and lift force coefficients

**Figure 5:**
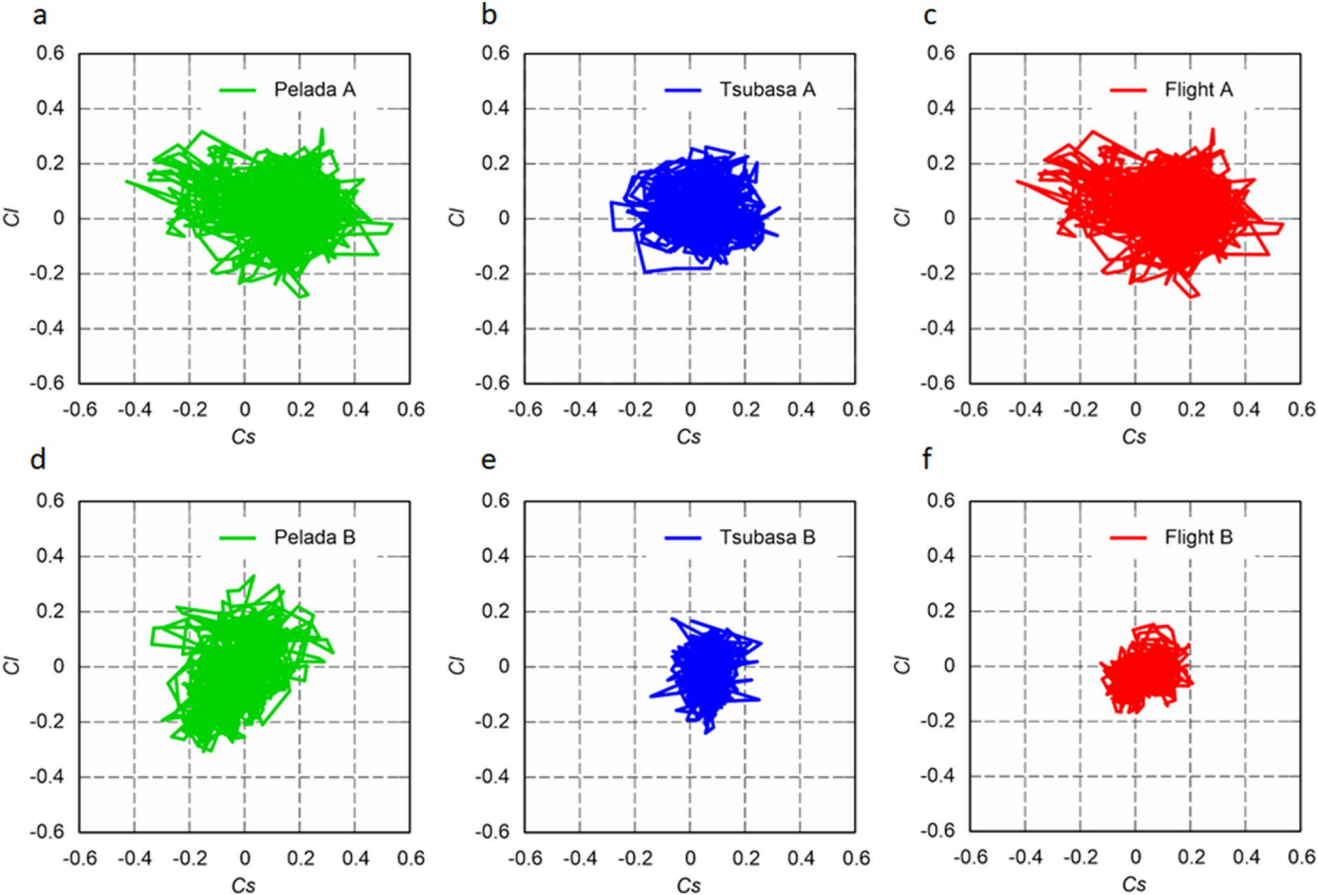
Scatter plots of the coefficients for the side force (*Cs*) and lift force (*Cl*) of Flight 2020, Tsubasa 2020, and Pelada 2020: (**a**–**c**) symmetric and (**d**–**f**) asymmetric orientations in the critical regime (initial ball speed of 15 m/s; Re = 2.1 × 10^5^).

**Figure 6:**
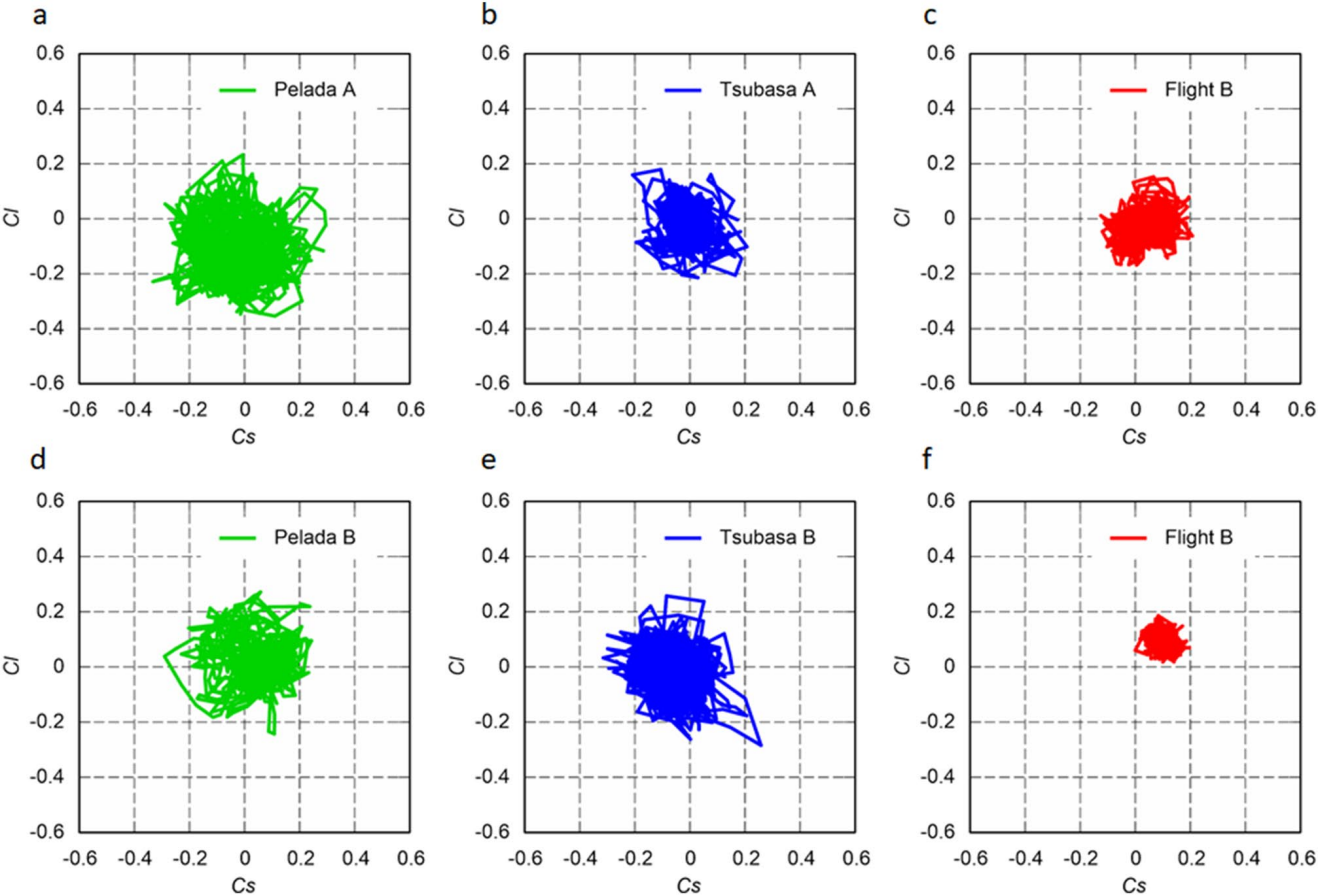
Scatter plots of the coefficients for the side (*Cs*) and lift (*Cl*) forces of Flight, Tsubasa, and Pelada: (**a**–**c**) symmetric and (**d**–**f**) asymmetric orientations in the supercritical regime (initial ball speed of 30 m/s; Re = 4.2 × 10^5^).

**Figure 7:**
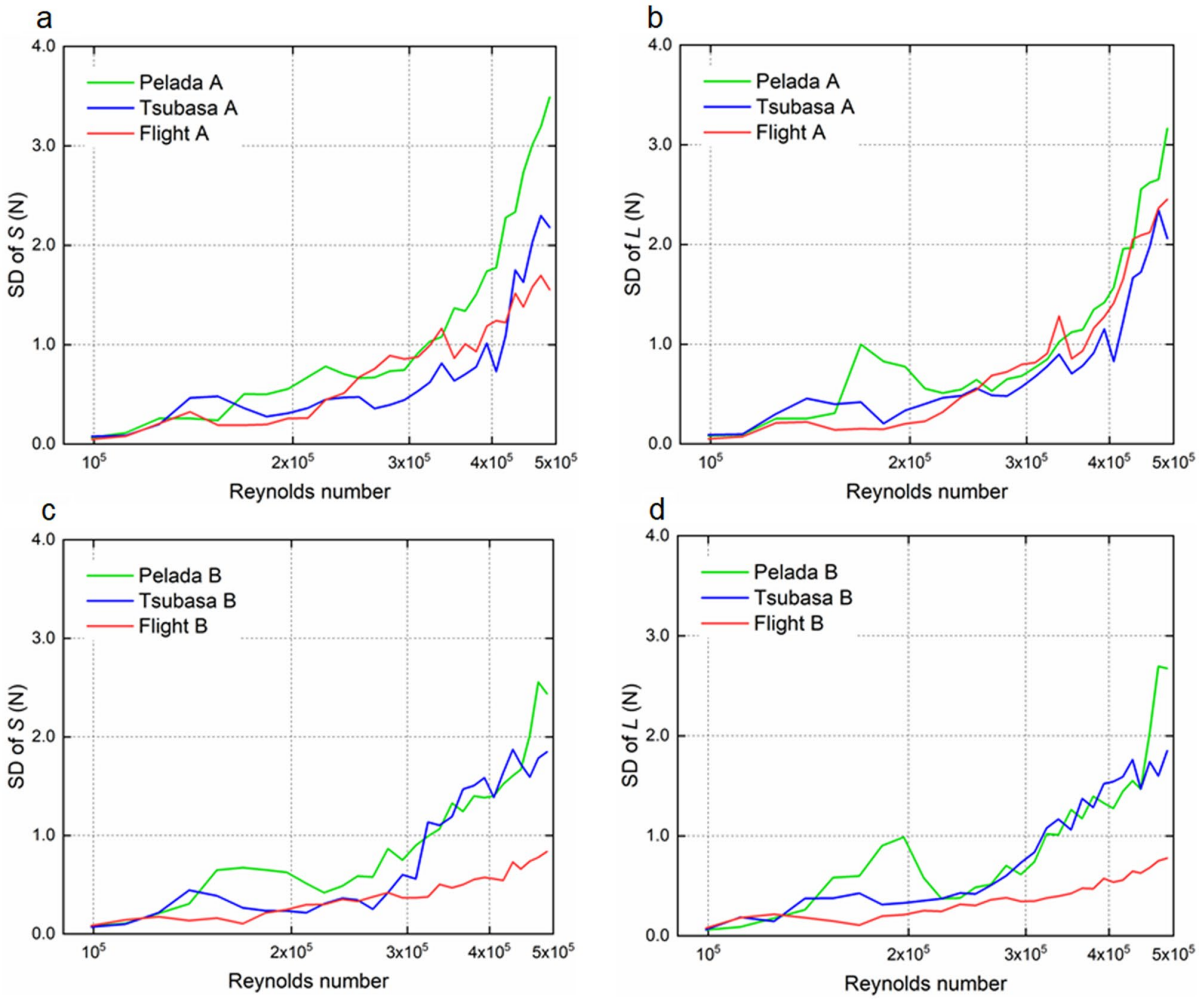
Standard deviation (SD) of the side (*S*) and lift (*L*) forces for Flight 2020, Tsubasa 2020, and Pelada 2020: (**a**,**b**) symmetric and (**c**,**d**) asymmetric orientations.

At an initial ball (flow) speed (*U*) of 15 m/s, Pelada 2020 exhibits large time-series fluctuations of the side force coefficient (*C*_*s*_) and lift force coefficient (*C*_*l*_), while Flight 2020 exhibits smaller time-series fluctuations (Fig. 5). Similar tendencies are demonstrated at an initial ball speed of 30 m/s (Fig. 6). The significance of the difference (*P* < 0.05) was determined through a multiple comparison test. The standard deviations of the side force (*S*) and lift force (*L*) tend to increase with speed for all balls and orientations (Fig. 7). The standard deviations of the side and lift forces are slightly smaller for Flight 2020 than for Pelada 2020. Moreover, the standard deviations of the side and lift forces are considerably smaller for Flight B than for Pelada B and Tsubasa B.

In the supercritical region, the average standard deviation of the side and lift coefficient are ~ 0.085 (s.d. = 0.015) for Pelada 2020, ~ 0.067 (s.d. = 0.015) for Tsubasa 2020, and ~ 0.054 (s.d. = 0.021) for Flight 2020. Statistically, the average standard deviations of the side and lift coefficient of Flight 2020 in the supercritical region tend to be significantly smaller than that of Pelada 2020 and Tsubasa 2020 (*p* < 0.001).

## Discussion

In the wind tunnel test, the smooth sphere has a critical Reynolds number of approximately 4.0 × 10^5^, which is in good agreement with the drag coefficient curve of Achenbach’s smooth sphere^16^ (Fig. 3). The critical Reynolds numbers of the 32-panel Pelada A and Pelada B are ~ 2.39 × 10^5^ (*C*_*d*_ ≈ 0.179) and ~ 2.52 × 10^5^ (*C*_*d*_ ≈ 0.153), respectively. These values do not perfectly agree with the critical Reynolds numbers (~ 2.5 × 10^5^ (*C*_*d*_ ≈ 0.16) and ~ 2.8 × 10^5^ (*C*_*d*_ ≈ 0.17)) of the 32-panel balls (Vantaggio, Molten Co.) investigated in our previous study^23^ because of the different panel materials. Meanwhile, Passmore et al*.*^15^ obtained a critical Reynolds numbers of ~ 2.5 × 10^5^ in the wind tunnel experiments using a 14-panel FIFA-approved ball. Kray et al*.*^32^ also reported critical Reynolds numbers in the range of 2.5 × 10^5^ measured from the wind tunnel experiments using a 14-panel ball. Hence, the aerodynamic coefficients obtained in this study agree reasonably well with previous experimental results.

In both the symmetric and asymmetric orientation cases, Flight 2020 has the lowest critical Reynolds number, followed by Tsubasa 2020 and then Pelada 2020. The critical Reynolds number of a spherical bluff body tends to decrease as the surface roughness increases^16^. The total length of the panel bonds and grooves, groove width, and groove depth on the surface has been suggested as an index of the surface roughness of a ball^23,24^. Notably, Flight 2020 has the widest groove width and longest total length, followed by Tsubasa 2020 and then Pelada 2020. These results indicate that Flight 2020 has the highest surface roughness, followed by Tsubasa 2020 and then Pelada 2020.

In both the symmetric and asymmetric orientation cases at the initial ball speed of 15 m/s (Re ≈ 2.10 × 10^5^), Flight 2020 has the longest flight distance than Pelada 2020. This is can be due to the initial ball speed, which is in the supercritical region for Flight 2020, where *C*_*d*_ is relatively low, whereas it is in the critical region for Pelada 2020, where *C*_*d*_ is increasing. In contrast, at an initial ball speed of 30 m/s (Re ≈ 4.20 × 10^5^), Flight 2020 has a shorter flight distance than Pelada 2020 in both the symmetric and asymmetric orientation cases. This may be attributed to the initial ball speed that is in the supercritical region for both balls. Hence, Flight 2020 has a shorter flight distance because it has a larger *C*_*d*_ than Pelada 2020.

The drag coefficients extracted by tracking a football in flight using high-speed video cameras and the subsequent trajectory analysis^18,33^ approximately confirmed the present drag coefficient curves obtained in the study. Flight A, which has a symmetrical ball face orientation, exhibits a substantially larger drag coefficient in the supercritical region compared to the other balls. Even in the ball trajectory simulation, the flight distance of Flight A is approximately 10% less than that of the other balls. Further, in the supercritical region, the average drag coefficient of Flight 2020 tends to be higher than that of the other balls (*p* < 0.001). Therefore, during its actual flight, where there is no rotation and the fluctuation of the stagnation point is extremely small, unlike in the wind tunnel experiment, it is suggested that Flight A could fly with higher air resistance than the other balls. However, this condition rarely occurs.

Based on the results, we concluded that Flight 2020 has a smaller critical Reynolds number and greater surface roughness than Pelada 2020, resulting in its slightly higher air resistance in the supercritical region. This may be attributed to the higher excitation occurring at the separation of the boundary layer on the surface of Flight 2020 owing to the presence of the panel bonds and grooves. Further, Flight 2020 exhibits smaller fluctuations (i.e. standard deviations) of its *C*_*s*_ and *C*_*l*_ values at initial ball speeds of 15 and 30 m/s than those of Pelada 2020 for both orientations. In addition, Pelada 2020 tends to have larger deviations of *S* and *L* than those of Flight 2020 at the different ball speeds.

The average deviations of the *S* and *L* of Flight 2020 are lower than that of the other balls in supercritical region (p < 0.001), which can be partially attributed to its higher surface roughness^23^. The deviations of *S* and *L* are inferred to be related to the instability of the flight trajectory without spinning or minimal spinning^21,22^. Therefore, Flight 2020 tends to suppress the instability of flight trajectory more than Pelada 2020 owing to its smaller deviations of *S* and *L*. In volleyball, Wei et al*.*^9^ found that the deviations of *S* and *L* are greater with the asymmetric orientation than the symmetric orientation. However, our results showed the opposite; the deviations tend to be smaller with the asymmetric orientation than with the symmetric orientation. This difference could be due to measurement region limited to the vicinity of the critical Reynolds number in the study of Wei et al*.*^9^, whereas a wider region from the subcritical to supercritical Reynolds numbers is considered in our study. Further, the different ball shapes are also another factor. In summary, Flight 2020 has a greater surface roughness and smaller critical Reynolds number than Pelada 2020, resulting to its marginally greater drag force in the supercritical region, and slightly smaller fluctuations of the side and lift forces.

Owing to the smaller critical Reynolds number of Flight 2020 than those of Tsubasa 2020 and Pelada 2020, it is expected to be travel slightly more for short passes (represented by the initial ball speed of approximately 15 m/s). However, because Flight 2020 has a large air resistance in the supercritical region, it should carry slightly less for strong shots, long free kicks, and other situations that required a longer distance (represented by the ball speed of approximately 30 m/s). Since Flight 2020 has smaller fluctuations in the side and lift forces than Tsubasa 2020 and Pelada 2020, it is expected to experience smaller irregular changes during the ball trajectory, thereby leading to its stability during flight.

This study has several limitations. First, we measured the aerodynamics of a non-spinning football fixed in a wind tunnel and did not take measurements using a spinning ball. However, in actual applications, the ball inevitably spins. Hence, the aerodynamics of a spinning ball needs to be considered. Second, the aerodynamic forces acting on the ball were measured, but the vortex structure around the ball and dynamics of the boundary layer that cause these forces have not been visualised or studied. These should also be examined through visualisation techniques, such as particle image velocimetry.

## Conclusions

This study aimed to clarify the aerodynamic characteristics (drag, side force, lift force, their deviations, and critical Reynolds number) of the new 4-panel ball (Flight 2020, Nike) in comparison to a 6-panel ball (Tsubasa 2020, Adidas) and conventional 32-panel ball (Pelada 2020, Molten) using a wind tunnel test, surface design measurement, and a simple 2D flight simulation.

Flight 2020 had a greater surface roughness and smaller critical Reynolds number than Pelada 2020 and Tsubasa 2020, which led to a marginally greater drag force in the supercritical region and slightly smaller fluctuations of the side and lift forces. In particular, the symmetrical orientation of Flight 2020 demonstrated a considerably higher drag coefficient in the supercritical region than the other studied balls, suggesting it could experience greater air resistance during flight under this condition. On the other hand, the smaller fluctuations in the side and lift forces of Flight 2020 than those of Pelada and Tsubasa 2020 indicates that it is more likely to experience smaller irregular changes in the ball trajectory, thereby possibly leading to its stability during flight.

## Data Availability

All data generated or analysed during this study are included in this published article.

## References

[1] Mehta RD (1985). Aerodynamics of sports balls. Annu. Rev. Fluid Mech..

[2] Watts RG, Ferrer R (1987). The lateral force on a spinning sphere: Aerodynamics of a curveball. Am. J. Phys..

[3] LeRoy W, Alaways LW, Hubbard M (2001). Experimental determination of baseball spin and lift. J. Sports Sci..

[4] Nathan AM, Hopkins J, Lance C, Hank K (2008). The effect of spin on the flight of a baseball. Am. J. Phys..

[5] Bray K, Kerwin DG (2003). Modelling the flight of a soccer ball in a direct free kick. J. Sports Sci..

[6] Passmore M, Spencer A, Tuplin S, Jones R (2008). Experimental studies of the aerodynamics of spinning and stationary footballs. Proc. Inst. Mech. Eng. P. J. Sports Eng. Technol..

[7] Bearman PW, Harvey JK (1976). Golf ball aerodynamics. Aeronaut. Q..

[8] Smith CE, Beratlis N, Balaras E, Squires K, Tsunoda M (2010). Numerical investigation of the flow over a golf ball in the subcritical and supercritical regimes. Int. J. Heat Fluid Flow.

[9] Wei Q, Lin R, Liu Z (1988). Vortex-induced dynamics loads on a non-spinning volleyball. Fluid Dyn. Res..

[10] Hong S, Ozaki H, Watanabe K, Asai T (2020). Aerodynamic characteristics of new volleyball for the 2020 Tokyo Olympics. Appl. Sci..

[11] Štěpánek A (1988). The aerodynamics of tennis balls: the topspin lob. Am. J. Phys..

[12] Zayas JM (1986). Experimental determination of the coefficient of drag of a tennis ball. Am. J. Phys..

[13] Mehta RD, Bentley K, Proudlove M, Varty P (1983). Factors affecting cricket ball swing. Nature.

[14] Asai T, Seo K, Kobayashi O, Sakashita R (2007). Fundamental aerodynamics of the soccer ball. Sports Eng..

[15] Passmore M (2012). The aerodynamic performance of a range of FIFA-approved footballs. Proc. Inst. Mech. Eng. P. J. Sports Eng. Technol..

[16] Achenbach E (1971). Influence of surface roughness on the cross-flow around a circular cylinder. J. Fluid Mech..

[17] Hong S, Asai T, Seo K (2015). Visualization of air flow around soccer ball using a particle image velocimetry. Sci. Rep..

[18] Goff JE, Carré MJ (2009). Trajectory analysis of a soccer ball. Am. J. Phys..

[19] Goff JE, Asai T, Hong S (2014). A comparison of Jabulani and Brazuca non-spin aerodynamics. Proc. Inst. Mech. Eng. P. J. Sports Eng. Technol..

[20] Goff JE, Hong S, Asai T (2019). Effect of a soccer ball’s seam geometry on its aerodynamics and trajectory. Proc. Inst. Mech. Eng. P. J. Sports Eng. Technol..

[21] Higuchi H, Kiura T (2012). Aerodynamics of knuckle ball: Flow-structure interaction problem on a pitched baseball without spin. J. Fluid. Struct..

[22] Murakami M, Seo K, Kondoh M, Iwai Y (2012). Wind tunnel measurement and flow visualization soccer ball knuckle effect. Sports Eng..

[23] Hong S, Asai T (2014). Effect of panel shape of soccer ball on its flight characteristics. Sci. Rep..

[24] Ward M, Passmore M, Spencer A, Tuplin S, Harland A (2019). Characterization of football trajectories for assessing flight performance. Proc. Inst. Mech. Eng. P. J. Sports Eng. Technol..

[25] Asai T, Seo K (2013). Aerodynamic drag of modern soccer balls. Springerplus.

[26] Naito K (2018). Effect of seam characteristics on critical Reynolds number in footballs. Mech. Eng. J..

[27] Asai T (2020). Soccer ball and flow. NAGARE.

[28] Fransson JHM, Matsubara M, Alfredsson PH (2005). Transition induced by free stream turbulence. J. Fluid Mech..

[29] Simoni D, Lengani D, Ubaldi M, Zunino P, Dellacasagrande M (2017). Inspection of the dynamic properties of laminar separation bubbles: free-stream turbulence intensity effects for different Reynolds numbers. Exp. Fluids.

[30] Blocken B, Persoon J (2009). Pedestrian wind comfort around a large football stadium in an urban environment: CFD simulation, validation and application of the new Dutch wind nuisance standard. J. Wind. Eng. Ind. Aerodyn..

[31] Sofotasiou P, Calautit J, Hughes B, O'Connor D (2016). Towards an integrated computational method to determine internal spaces for optimum environmental conditions. Comput. Fluids.

[32] Kray T, Franke J, Frank W (2014). Magnus effect on a rotating soccer ball at high Reynolds numbers. J. Wind Eng. Ind. Aerodyn..

[33] Goff JE, Smith WH, Carré MJ (2011). Football boundary layer separation via dust experiments. Sports Eng..

